# Absence of the Lectin Activation Pathway of Complement Ameliorates Proteinuria-Induced Renal Injury

**DOI:** 10.3389/fimmu.2019.02238

**Published:** 2019-09-23

**Authors:** Samy Alghadban, Hany I. Kenawy, Thomas Dudler, Wilhelm J. Schwaeble, Nigel J. Brunskill

**Affiliations:** ^1^Department of Infection, Immunity and Inflammation, College of Life Sciences, University of Leicester, Leicester, United Kingdom; ^2^Zoology Department, Faculty of Science, Al-Azhar University, Cairo, Egypt; ^3^Microbiology and Immunology Department, Faculty of Pharmacy, Mansoura University, Mansoura, Egypt; ^4^Omeros Corporation, Seattle, WA, United States; ^5^Department of Veterinary Medicine, University of Cambridge, Cambridge, United Kingdom; ^6^Department of Nephrology, Leicester General Hospital, Leicester, United Kingdom

**Keywords:** lectin pathway, proteinuria, kidney, MASP, MBL-associated serine proteases, nephropathy

## Abstract

Proteinuria is an adverse prognostic feature in renal diseases. In proteinuric nephropathies, filtered proteins exert an injurious effect on the renal tubulointerstitium, resulting in inflammation and fibrosis. In the present study, we assessed to what extent complement activation via the lectin pathway may contribute to renal injury in response to proteinuria-related stress in proximal tubular cells. We used the well-established mouse model of protein overload proteinuria (POP) to assess the effect of lectin pathway inhibition on renal injury and fibrotic changes characteristic of proteinuric nephropathy. To this end, we compared experimental outcomes in wild type mice with MASP-2-deficient mice or wild type mice treated with MASP-2 inhibitor to block lectin pathway functional activity. Multiple markers of renal injury were assessed including renal function, proteinuria, macrophage infiltration, and cytokine release profiles. Both MASP-2-deficient and MASP-2 inhibitor-treated wild type mice exhibited renoprotection from proteinuria with significantly less tubulointerstitial injury when compared to isotype control antibody treated mice. This indicates that therapeutic targeting of MASP-2 in proteinuric nephropathies may offer a useful strategy in the clinical management of proteinuria associated pathologies in a variety of different underlying renal diseases.

## Introduction

Proteinuria is a risk factor for the progressive decline of renal excretory function, and the development of renal fibrosis regardless of the primary renal disease (1–5). The concept of proteinuric nephropathy describes the toxic effects of excess protein entering the proximal tubule as a result of the impaired glomerular permeability (6, 7). This phenomenon, common to many glomerular diseases, results in a pro-inflammatory and pro-fibrotic environment in the kidney and is characterized by alterations in proximal tubular cell growth, apoptosis, gene transcription, and inflammatory cytokine production due to dysregulated signaling pathways stimulated by proteinuric tubular fluid. Proteinuria is generally recognized to be a key contributor to progressive renal injury common to diverse primary renal pathologies.

Complement activation has been widely described in immune complex-mediated and other glomerulonephritis, but several investigators have also demonstrated an important role for complement in the development of tubulointerstitial (TI) fibrosis in non-glomerular disease (8–10). This may result from complement components filtered into the proximal tubule, abnormal synthesis of complement components by tubular cells or other resident or infiltrating cells, or altered expression of complement regulatory proteins by kidney cells (10, 11). In mice, deficiency of the complement regulatory protein CR1-related gene/protein y (Crry) results in TI complement activation with consequent inflammation and fibrosis typical of the injury seen in human TI diseases (9–12). Exposure of tubular epithelial cells to the anaphylatoxin C3a results in epithelial to mesenchymal transition (13). Blocking C3a signaling through the C3a receptor prevents this and lessens renal TI fibrosis in proteinuric and non-proteinuric animals (13, 14), demonstrating the potential for therapeutic targeting of complement activation in renal TI disease. Given the paucity of new and existing treatments targeting inflammatory and pro-fibrotic pathways in renal disease, new avenues of therapeutic intervention deserve closer attention.

Complement activation via the lectin pathway (LP) is initiated by the binding of LP-specific pattern recognition subcomponents. Two types of LP-recognition subcomponents have been described: Collectins [including Mannan binding lectin (MBL), Collectin-11 (CL-11), and Collectin-11/collectin-10 heterotrimers] which can bind to carbohydrate patterns and ficolins that bind to acetylated ligand patterns on pathogens and injured cells (15–17). All LP-pattern recognition subcomponents can form complexes with 3 different LP-associated enzymes (called MBL-associated serine proteases MASP-1, MASP-2, and MASP-3). Binding of LP-recognition complexes to their cognate ligands converts MASP zymogens into their enzymatically active form to drive LP-mediated complement activation. In this process, MASP-2 is the critical enzyme to generate the LP-C3 convertase C4b2a, since it is the only MASP that can cleave both complement C2 and C4 (18, 19). The absence of MASP-2 results in the total loss of LP-mediated complement activation (20).

LP-driven complement activation is part of the innate immune defense. However, LP-mediated complement activation has also been implicated in the pathophysiology of a diverse range of renal diseases including IgA nephropathy and other glomerulonephritis (21–25), diabetic nephropathy (26), ischemia reperfusion injury (27), and transplant rejection (28).

To establish the role of the lectin pathway in renal TI inflammation, we studied the effect of MASP-2 gene ablation on experimental outcomes using the well-characterized model of protein overload proteinuria (POP). In addition, the effect of treatment with an inhibitory MASP-2 antibody was evaluated in wild-type mice and compared to an isotype-matched control antibody. The results demonstrate a salutary effect of MASP-2 inhibition on renal TI inflammation, tubular cell injury, pro-fibrotic cytokine release, and scarring.

## Materials and Methods

### Experimental Animals

All animal procedures were subject to institutional ethical review and approved under UK Home Office Project License PPL 60/4438. Male BALB/c mice, either wild type (WT) or MASP-2^−/−^ [please see reference (20) for a detailed description of this mouse model and its phenotype], were kept under standardized conditions of 12 h day/12 h night light cycle, with standard diet and *ad libitum* access to water. Animals were acclimatized for at least 1 week before experimental procedures and housed 5/cage in a pathogen-free environment.

For POP, 8 week-old mice first underwent left nephrectomy via a short flank incision and were allowed to recover for 7 days before starting bovine serum albumin (BSA) injections. To induce POP, mice were given daily intraperitoneal (IP) injections of BSA (low endotoxin, filter-sterilized 400 mg/mL solution of BSA (Sigma cat# A4919) in saline), starting from 2 mg/g on day one and increasing to 15 mg/g of body weight on day 7, followed by an additional 7 daily doses of 15 mg/g. After the last injection on day 14, animals were placed in metabolic cages for 24 h urine collections. Mice were sacrificed 24 h after the last BSA administration. To inhibit LP, WT mice with POP were treated with HG4, a MASP-2-specific mAb optimized for LP inhibition in mice (29). HG4 or isotype-matched control antibody was administered by ip injection at a dose of 10 mg/kg to groups of 8 mice. Saline-injected mice were included as an additional control group. Mice were dosed bi-weekly, starting 7 days before proteinuria induction and continuing throughout the study. Blood was taken prior to each dose and at the end of the experiment to assess LP-functional activity.

At sacrifice, blood was collected by cardiac puncture under anesthesia, various tissues were collected for analysis and the mice sacrificed by exsanguination. Blood was allowed to clot on ice for 2 h, serum was then collected by centrifugation and stored in aliquots at −80°C.

### Biochemical Analyses

Serum and urine creatinine concentrations were measured using the QuantiChrom assay kit (BioAssay Systems, Hayward, CA). Urine and serum total proteins were measured using the Coomassie Plus™ Assay Kit (Fisher Scientific, Loughborough, UK).

### Analysis of Kidney Tissue

Kidney morphology was assessed by light microscopy of haematoxylin- and eosin (H&E)-stained sections of formalin-fixed, paraffin-embedded (FFPE) kidney tissue. Collagen deposition was evaluated in 5-μm sections of FFPE kidney tissue by staining with picrosirius red as previously described (30). To detect infiltrating macrophages by immunohistochemistry (IHC), 5-μm FFPE kidney sections were deparaffinized and rehydrated. Antigen retrieval was performed in citrate buffer and endogenous peroxidase activity quenched by incubation in 3% H_2_O_2_ for 10 min. Tissue sections were incubated in blocking buffer (10% heat-inactivated normal goat serum with 1% BSA in PBS) for 1 h at room temperature followed by avidin/biotin blocking. Sections were washed 3 × 5 min in PBS after each step. An F4/80 anti-macrophage antibody (Santa Cruz, Dallas, TX), diluted 1:100 in blocking buffer, was applied for 1 h. A biotinylated goat anti-rat secondary antibody, diluted 1:200 in blocking buffer, was then applied for 30 min followed by HRP conjugate enzyme for 30 min. Staining was developed using diaminobenzidine (DAB) substrate (Vector labs, Peterborough, UK) for 10 min and slides were washed in water, dehydrated, and mounted without counter-staining to facilitate the computer-based image analysis.

Apoptotic cells in FFPE kidney sections were detected using an ApopTag® Kit (Millipore, Watford, UK) according to the manufacturer's instructions. For apoptosis experiments, technical negative controls lacked anti-digoxigenin antisera. Tissue sections were counterstained with haematoxylin. Brown-colored apoptotic cells were counted manually in 20 adjacent non-overlapping cortical high-power fields. Proinflammatory and profibrotic cytokines were studied by IHC in 5-μm sections of FFPE kidney. Antigen retrieval was performed in citrate buffer followed by quenching of endogenous peroxidase activity with 3% H_2_O_2_ for 10 min. Sections were then incubated in blocking buffer with 10% avidin solution for 1 h at room temperature. Sections were washed for 5 min x3 in PBS after each step. Primary antibodies were applied in blocking buffer with 10% biotin solution for 1 h at a concentration of 1:100 for the antibodies anti-TGFβ1 (Santa Cruz, cat# sc-7892), and anti-IL-6 (Santa Cruz, cat# sc-1265), and at 1:50 for the anti-TNFα antibody (Santa Cruz, cat# sc-1348). Biotinylated secondary antibodies were then applied for 30 min followed by HRP conjugate enzyme for another 30 min. Color was developed using DAB substrate kit (Vector Labs) for 10 min and slides were washed in water, dehydrated, and mounted without counter-staining to facilitate computer-based image analysis. For IHC experiments, technical negative controls lacked primary antisera.

For electron microscopy, kidney cortex was fixed in 2% glutaraldehyde in PBS. Tissue samples were post-fixed in aqueous osmium tetroxide and were dehydrated and embedded in epoxy resin. Ultrathin sections (70–90 nm) were cut on a Reichart-Jung OMU-4 ultramicrotome, contrasted with uranyl nitrate and lead citrate, and examined with a JEOL 100CX electron microscope.

### Image Analysis

The percentage-stained area of kidney cortex was measured as previously described (31). Briefly, 24-bit color images were captured from sequential non-overlapping fields of renal cortex just beneath the renal capsule around the entire periphery of the kidney section. After each image capture, NIH Image was used to extract the red channel as an 8-bit monochrome image. Unevenness in the background illumination was subtracted using a pre-recorded image of the illuminated microscope field with no section in place. The image was subjected to a fixed threshold to identify areas of the image corresponding to the staining positivity. The percentage of black pixels was then calculated and, after all the images around the kidney had been measured, the mean percentage was recorded, providing a figure corresponding to the percentage of antibody-binding area in the kidney section. A set of macros was written to automate this process, such that assessing one kidney section typically took 10 min.

### Quantification of C3c Deposition Using Enzyme Linked Immunosorbent Assay (ELISA)

To assess LP functional activity in mouse serum, the deposition of the C3 activation (cleavage) product C3c was measured by ELISA as described (20). Briefly, microtitre plates were coated with 1 μg/well of mannan diluted in coating buffer (15 mM Na_2_CO_3_, 35 mM NaHCO_3_, pH 9.6) overnight at 4°C, then blocked using 5% skimmed milk in TBS buffer (10 mM Tris-Cl, 140 mM NaCl, pH 7.4) Serum samples from individual mice stored at −80°C were slowly thawed on ice, a 2.5% serum dilution from each sample was prepared in barbital-buffered saline (BBS; 4 mM barbital, 145 mM NaCl, 2 mM CaCl_2_, 1 mM MgCl_2_, pH 7.4), and 100 μL of diluted serum samples were added to wells of the Mannan-coated assay plate. Negative control wells had only BBS buffer. After incubation of the assay plates at 37°C for 1 h, plates were washed x4 in TBS/TW/Ca^++^ buffer (TBS with 0.05% Tween 20 and 5 mM CaCl_2_). Bound C3c was detected with a polyclonal anti-human-C3c antibody (Dako, Santa Clara, CA, cat# A0062) diluted in TBS/TW/Ca^++^ buffer at 1:5,000, incubated at 4°C overnight. After washing x4, alkaline phosphatase-conjugated goat anti-rabbit IgG, 1:10,000 in TBS/TW/Ca^++^ was added and incubated for 1 h at room temperature. C3c deposition was quantified following addition of 100 μL of Sigma Fast *p*-nitrophenyl phosphate substrate solution (Sigma-Aldrich) and incubation at room temperature. The absorption (OD) of the developed color was measured at 405 nm after 8 min.

### Data Analysis

All data are presented as mean ± SEM of at least *n* = 3 experiments. Statistical analyses were performed by ANOVA using the Bonferroni *post-hoc* test where appropriate.

## Results

**Figure 1:**
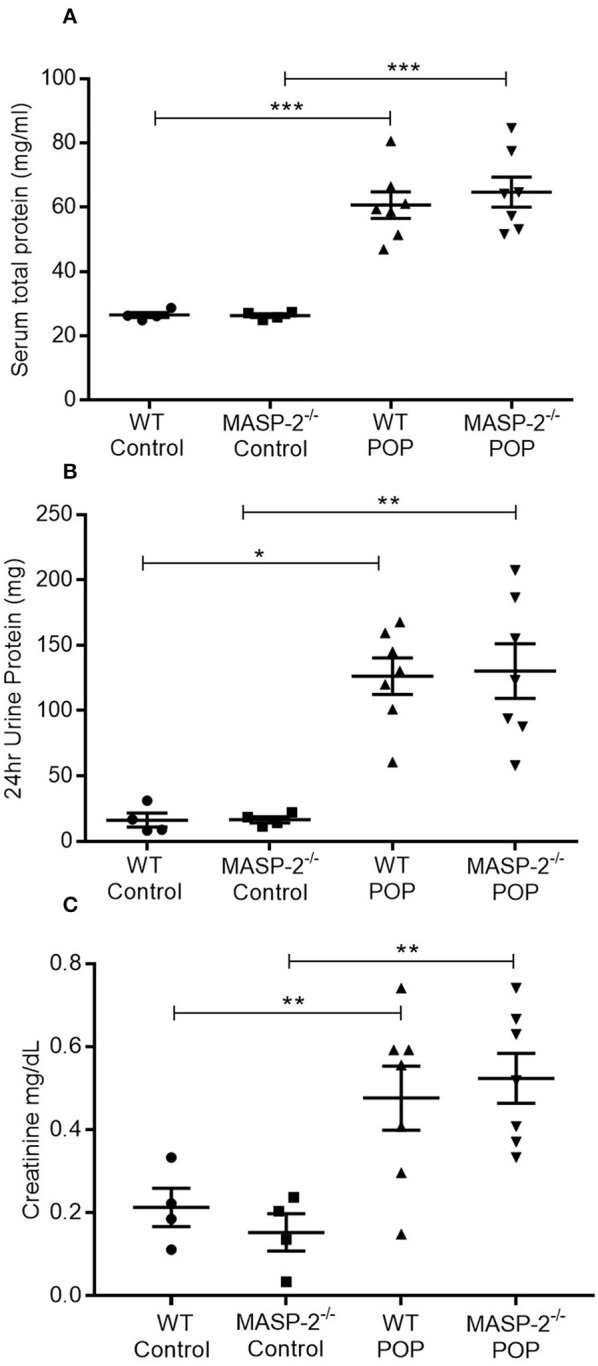
Urine and serum parameters in mice with POP. WT and MASP-2^−/−^ mice were either left untreated (control) or subject to POP as described. **(A)** Serum total protein concentrations in control mice and mice with POP; **(B)** 24 h urine protein excretion in mice with POP; **(C)** serum creatinine levels in control mice and mice with POP. In all graphs, individual symbols represent values from individual animals measured in duplicate. ^*^p < 0.05, ^**^p < 0.01, ^***^p < 0.001 by one-way ANOVA with Bonferroni *post-hoc* test for multiple comparisons.

The role of the LP in renal TI injury was studied in WT and MASP-2^−/−^ mice with POP. In response to protein overload, WT mice developed significant proteinuria in association with elevated serum protein levels as well as significant elevations in serum creatinine (Figure 1) as previously described (32). Comparable changes were observed in MASP-2^−/−^ mice (Figure 1).

**Figure 2:**
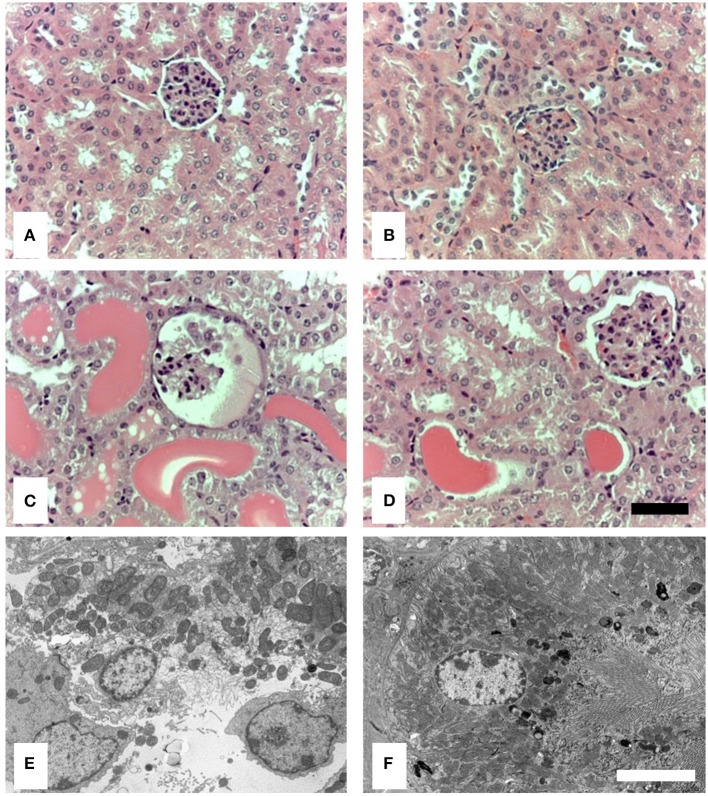
Histological and ultrastructural appearance of kidneys from control mice and mice with POP. Kidneys from mice were sectioned and stained with H&E or prepared for electron microscopy (EM). **(A)** Represents H&E stained sections from untreated WT. **(B)** Represents H&E stained sections from untreated MASP-2-deficient mice. **(C)** Depicts H&E stained sections from proteinuric WT mice, whereas **(D)** depicts H&E stained sections from proteinuric MASP-2-deficient mice. **(E)** Represents sections examined by EM from proteinuric WT mice. **(F)** Represents sections examined by EM from proteinuric MASP-2-deficient mice. In H&E sections scale bar = 100 μm and in electron micrographs scale bar = 5 μm.

By light microscopy (LM), there was no evident difference in renal histology between control-treated wild-type and MASP-2^−/−^ mice (Figures 2A,B). In proteinuric wild-type animals, there was clear proximal tubular (PT) cell vacuolation, tubular dilatation, and multiple tubular protein casts (Figure 2C). These abnormalities were less marked in MASP-2^−/−^ mice (Figure 2D). By electron microscopy (EM), wild-type animals with proteinuria showed lysed PT cells with apical blebbing, disrupted brush borders, and cell contents in tubular lumina (Figure 2E), whereas in proteinuric MASP-2^−/−^ animals, PT structure was largely preserved (Figure 2F). These data suggest reduced tubulointerstitial injury in response to proteinuria in MASP-2-deficient compared to WT mice.

**Figure 3:**
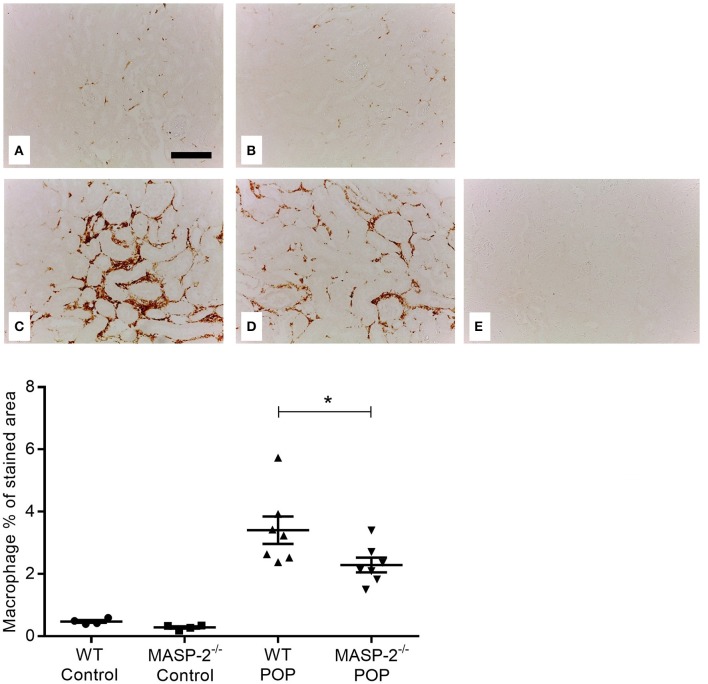
Macrophage infiltration in kidneys of mice with POP. Photomicrographs represent kidneys from animals with POP stained for macrophages. **(A)** Non-proteinuric wild type (WT) controls; **(B)** non-proteinuric MASP-2^−/−^ controls; **(C)** proteinuric WT mice with POP; **(D)** proteinuric MASP-2^−/−^ mice with POP; **(E)** negative control lacking primary antibody in a section from non-proteinuric WT mice Scale bar = 100 μm. Graph depicts quantification of macrophage infiltration in POP mice as described in Methods. Each data point represents values from an individual animal. ^*^p < 0.05, by one-way ANOVA with Bonferroni *post-hoc* test for multiple comparisons.

To extend these findings, kidney macrophage infiltration in response to POP was studied. Proteinuric MASP-2^−/−^ mice had significantly fewer macrophages in their renal tubulointerstitium compared to their respective proteinuric WT controls (Figure 3).

**Figure 4:**
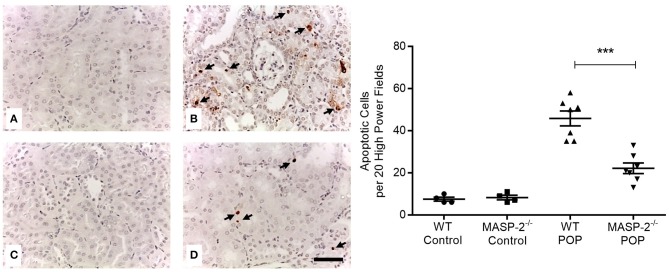
Cell apoptosis in kidneys of mice with POP. Apoptotic cells in mouse kidneys were identified by TUNEL staining as per Methods. **(A)** Wild type (WT) control mice; **(B)** proteinuric WT mice with POP; **(C)** MASP-2^−/−^ control mice; **(D)** proteinuric MASP-2^−/−^ mice with POP. Arrows indicate examples of apoptotic cells, scale bar = 100 μm. Graph depicts quantification of apoptotic cells with each data point derived from an individual animal. ^***^p < 0.0001 by one-way ANOVA with Bonferroni *post-hoc* test for multiple comparisons.

Moreover, proteinuria was associated with significantly increased apoptosis of PT and other TI cells (Figure 4). This apoptosis was significantly less prominent in MASP-2^−/−^ mice compared to their wild-type controls (Figure 4).

**Figure 5:**
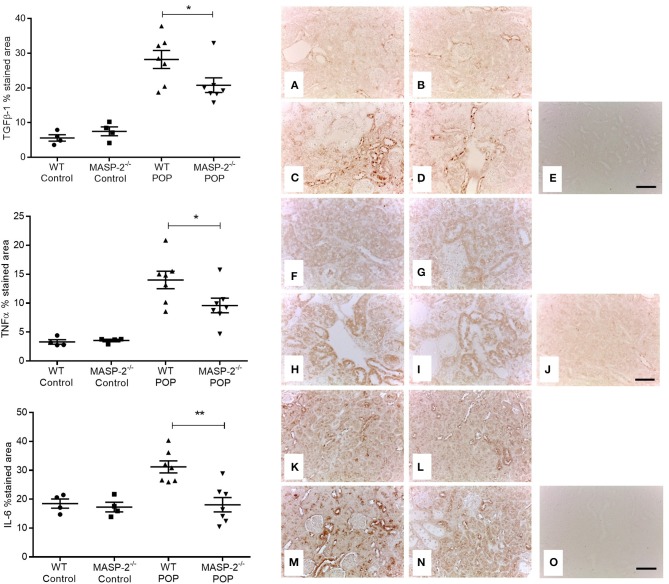
Investigation of mouse kidneys for inflammatory and pro-fibrotic mediators. IHC was performed for **(A–E)** TGFβ1; **(F–J)** TNFα; and **(K–O)** IL-6, in **(A,F,K)** depict wild type (WT) control mice; **(B,G,L)** depict MASP-2^−/−^ control mice; **(C,H,M)** depict WT mice with POP; and **(D,I,N)** depict MASP-2^−/−^ mice with POP; **(E,J,O)** depict negative controls where sections from WT mice lack primary antibody. Scale bar = 100 μm. Quantification of IHC staining is shown in graphs alongside their relevant IHC panels. Each data point is derived from an individual animal. ^*^p < 0.05, ^**^p < 0.01 by one-way ANOVA with Bonferroni *post-hoc* test for multiple comparisons.

The expression of several inflammation- and fibrosis-related proteins and genes was determined in animals with POP. In proteinuric mice, expression of TGFβ1, TNFα, and IL-6 were significantly increased as shown by IHC in renal cortex compared to non-proteinuric controls, but this enhancement of expression was attenuated in proteinuric MASP-2^−/−^ mice compared to proteinuric WT mice (Figure 5). The abundance of collagen alpha-1(IV) chain mRNA was significantly elevated in proteinuric WT mice (0.35 ± 0.06 Log_10_ RQ) compared to proteinuric MASP-2^−/−^ mice (0.14 ± 0.06 Log_10_ RQ, = *p* < 0.05 ANOVA with Bonferroni correction), however there was no difference in collagen protein deposition detectable in kidneys by picrosirius red staining. Expression levels of genes for TGFβ1, TNFα, IL-6, interferon-γ, properdin, and complement C3 were not significantly different between proteinuric WT and proteinuric MASP-2^−/−^ mice (Supplementary Figure 1).

**Figure 6:**
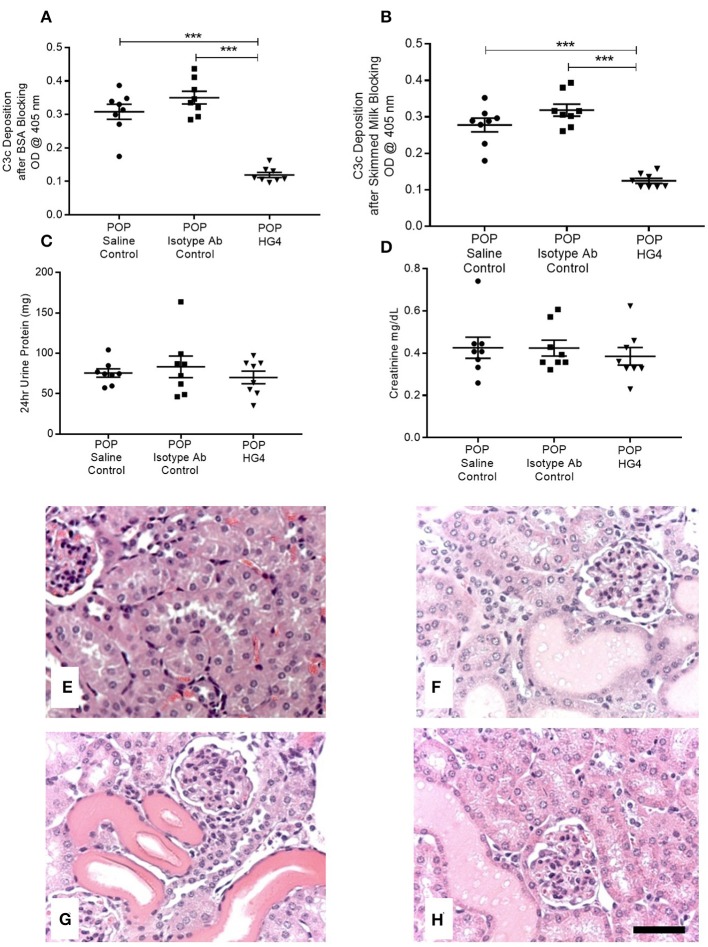
Deposition of C3c as a measure of serum lectin pathway activity, serum creatinine levels, urine protein excretion, and histopathology in control and antibody treated POP WT mice. **(A,B)** Depict residual serum LP activity in each group of animals measured as deposition of C3c by ELISA using **(A)** BSA blocking buffer; **(B)** skimmed milk blocking buffer. Graphs **(C,D)** represent 24 h urine protein excretion and serum creatinine, respectively, in proteinuric animals injected with saline, isotype control antibodies, or HG4 antibodies as labeled. Each data point depicts serum analysis from an individual animal. ^***^p < 0.0001 by one-way ANOVA with Bonferroni *post-hoc* test for multiple comparisons. **(E–H)** Kidneys from mice were sectioned and stained with H&E. **(E)** WT control, **(F)** saline injected proteinuric WT mice; **(G)** isotype control antibody injected proteinuric WT mice; **(H)** HG4 injected proteinuric WT mice. Scale bar = 100 μm.

**Figure 7:**
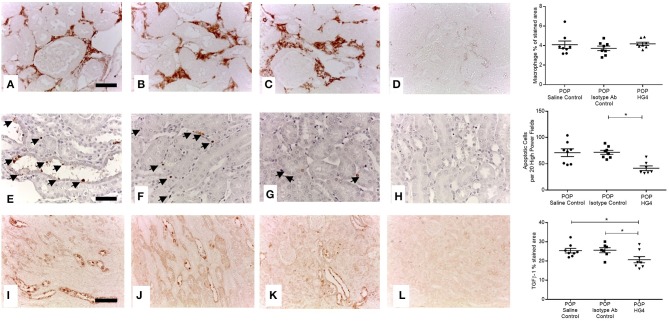
Tubulointerstitial macrophage infiltration, apoptosis and TGFβ1 expression in antibody treated and control WT mice with POP. Macrophage infiltration (**A–D** scale bar = 100 μm), cell apoptosis (**E–H** scale bar = 50 μm), and TGFβ1 protein expression (**I–L** scale bar = 100 μm) were assessed and quantified according to Methods. **(A,E,I)** Saline injected POP mice; **(B,F,J)** isotype control injected POP mice and; **(C,G,K)** HG4 antibody injected POP mice; **(D,H,L)** negative control sections from control WT mice lacking primary antibody or anti-digoxigenin antibody in the case of apoptosis detection. Graphs depict quantification of macrophage staining, numbers of apoptotic cells, or quantification of TGFβ1 staining. Each data point relates to an individual animal. For macrophage and apoptosis quantification ^*^p < 0.01, and for TGFβ1 quantification ^*^p < 0.05 both by one-way ANOVA with Bonferroni *post-hoc* test for multiple comparisons.

To extend these findings, we assessed the effect of treatment with the rodent specific anti-MASP-2 antibody HG4 on POP-related pathological changes in WT mice. We first verified that administration of HG4 to WT mice with POP mice blocked LP functional activity. While serum samples of both saline and isotype control treated POP mice showed robust lectin-induced C3c deposition, minimal residual C3c deposition was observed in serum samples from HG4-treated mice, indicating effective inhibition of LP functional activity following treatment of POP mice with this antibody (Figures 6A,B). There were no significant differences in serum creatinine and proteinuria between saline control, isotype control, and HG4-treated POP mouse groups during the short observation period (Figures 6C,D). As with the studies in MASP-2^−/−^ mice, H&E sections of kidneys from proteinuric HG4-treated animals revealed less renal injury resulting in reduced PT cell vacuolation, less tubular dilatation and less tubular protein cast formation (Figures 6E–H). Inhibition of LP with HG4 had no significant effect on kidney macrophage infiltration (Figures 7A–D) but did significantly reduce TI apoptosis in proteinuric WT mice (Figures 7E–H). Furthermore, HG4 significantly reduced TGFβ1 protein expression in the tubulointerstitium of kidneys from proteinuric WT mice (Figures 7I–L). Also, by IHC, HG4 reduced the expression of TNFα in the kidneys of proteinuric WT mice compared to saline injected WT controls with proteinuria (14.36 ± 0.57 vs. 16.97 ± 0.68, respectively; *n* = 8, *p* < 0.05, ANOVA with Bonferroni correction for multiple comparisons, micrographs not shown). mRNA expression levels for TGFβ1, TNFα, IL-6, interferon-γ, properdin, collagen alpha-1(IV), and complement factor C3 were not significantly different between isotype control and HG4-treated proteinuric WT mice (Supplementary Figure 2).

## Discussion

Proteinuria is a powerful predictor of renal disease progression and functional decline (4, 5, 33). Because there is a strong biological rationale for a causal link between proteinuria and renal TI inflammation and fibrosis (6, 7), novel interventions targeting proteinuria-mediated TI inflammation and fibrosis hold promise for the treatment of a variety of renal conditions associated with proteinuria. The current studies using a well-established mouse model of proteinuria demonstrate that inhibiting the LP of complement activation by either genetic modification or pharmacologic intervention using specific antibodies blocking MASP-2 function leads to abrogation of renal injury without altering levels of experimentally induced proteinuria. The results suggest that modulating LP activity by blocking MASP-2 function reduces the harmful effects of proteinuria. Because in the context of proteinuria-induced renal injury the absence or presence of membrane attack complex (MAC) was recently shown to have no influence on the severity of TI injury and renal impairment within the time frame of our experiments, MAC deposition was not used as a parameter to assess injury or as a prognostic indicator of disease severity in the current studies (34).

POP is a well-established rodent model of minimal change disease. Mice display strain-related differences in their response to protein overload where some mice are relatively resistant to the development of proteinuria. BALB/c mice were used in these studies because they exhibit a response to protein overload that most closely matches the phenotype of human kidney disease (32, 35, 36). In the current study, the POP phenotype is comparable with that described previously and manifest as prompt proteinuria and a modest rise in serum creatinine (32, 35, 36). POP mice display early TI inflammation, but fibrosis is only evident at a later time point than that used in our studies (35). Severity limits of our animal license precluded the continuation of POP beyond the 2-week point used in the current studies.

In proteinuric MASP-2^−/−^ mice, absent LP activity was associated with broad evidence of renoprotection in the form of improved histological appearances, reduced macrophage infiltration, reduced apoptosis, and reduced production of deleterious mediators of inflammation and scarring. Taken together our findings indicate that MASP-2 inhibition may be broadly protective in proteinuric nephropathy.

Targeting the MASP-2 serine protease with an inhibitory mAb effectively blocks the LP of complement activation, whilst leaving the functional activities of the two other complement pathways intact (20). Absence of LP activity in MASP-2 gene-targeted mice and in WT mice treated with MASP-2 inhibitors confers significant protection from ischemia/reperfusion injury in mouse models of myocardial, intestinal and cerebral ischemia (20, 29, 37). In the current studies we confirmed that administration of the MASP-2-blocking antibody HG4 effectively inhibited LP in POP mice for the duration of the study. In mice with POP, inhibition of MASP-2 is strongly associated with renoprotection exemplified by improved histological appearances of proteinuric kidneys, reduced TI apoptosis, and reduced expression of TGFβ1. Unlike in proteinuric MASP-2^−/−^ mice no significant effect of HG4 on renal macrophage infiltration was observed in proteinuric WT mice. We are currently refining HG4 dosage schedules in mice to ensure optimum inhibition of MASP-2 within the kidney.

Cytokine studies also indicate renoprotection associated with inhibition of MASP-2. Multiple cytokines have been implicated in the pathophysiology of proteinuria-induced TI inflammation (7, 38), and several of these were evaluated in the current studies. Amongst these, TGFβ1 plays a key role in the development of renal fibrosis and was reduced at the protein level in both MASP-2^−/−^ and HG4 treated mice with proteinuria. Protein expression of some other pathogenic cytokines was also reduced in MASP-2^−/−^ mice with proteinuria.

Under conditions of ischemic stress in the kidney, aberrant basolateral expression of L-fucose by renal tubules is associated with enhanced proximal tubular expression of collectin-11, which in turn triggers LP activation via MASP-2 at the basolateral membrane (39). While MASP-2 is exclusively synthesized in the liver, the local biosynthesis of the LP recognition molecule collectin-11 can be induced by ischemic or hypothermic stress in proximal tubular cells. The renal biosynthesis of CL-11 appears to be critical, since transplanted kidneys of collectin-11-deficient mice into wildtype mice are protected from renal fibrosis after ischemia-reperfusion (40). The stress-induced *de novo* biosynthesis of collectin-11 in the kidney further underscores the importance the LP in the development of renal inflammation in ischaemia-reperfusion (39). Substituting ischemic stress with proteinuric stress mediated by filtered proteins across the apical membrane of PT cells may result in a similar response.

In summary, this study demonstrates that the LP plays a central role in the development of renal injury in proteinuria and that inhibiting MASP-2 favorably modulates multiple key renal pathophysiological abnormalities resulting from proteinuria. Thus, novel therapies based on targeting MASP-2 as the essential enzyme of the LP may provide a promising new approach to treat chronic proteinuric nephropathies regardless of the underlying etiology. Further studies will be necessary to determine the optimal treatment modality to achieve a significant improvement in the clinical outcomes in proteinuric kidney disease.

## Data Availability Statement

The datasets generated for this study are available on request to the corresponding author.

## Ethics Statement

All animal procedures were subject to institutional ethical review and approved under UK Home Office Project License PPL 60/4438.

## Author Contributions

The studies were conceived and designed by NB and WS. All *in vivo* work was performed by SA and HK who also prepared and analyzed data. TD supplied the antibody for intervention studies. NB wrote the initial manuscript. All authors contributed to data interpretation and read and edited the manuscript.

### Conflict of Interest

TD is an employee and shareholder of Omeros Crop. WS is a shareholder of Omeros Corp. The remaining authors declare that the research was conducted in the absence of any commercial or financial relationships that could be construed as a potential conflict of interest.
