# Supplementary figures and images for: Absence of the Lectin Activation Pathway of Complement Ameliorates Proteinuria-Induced Renal Injury
Source: Front Immunol. 2019 Sep 23;10:2238. doi: 10.3389/fimmu.2019.02238 (PMC6768126; doi:10.3389/fimmu.2019.02238)

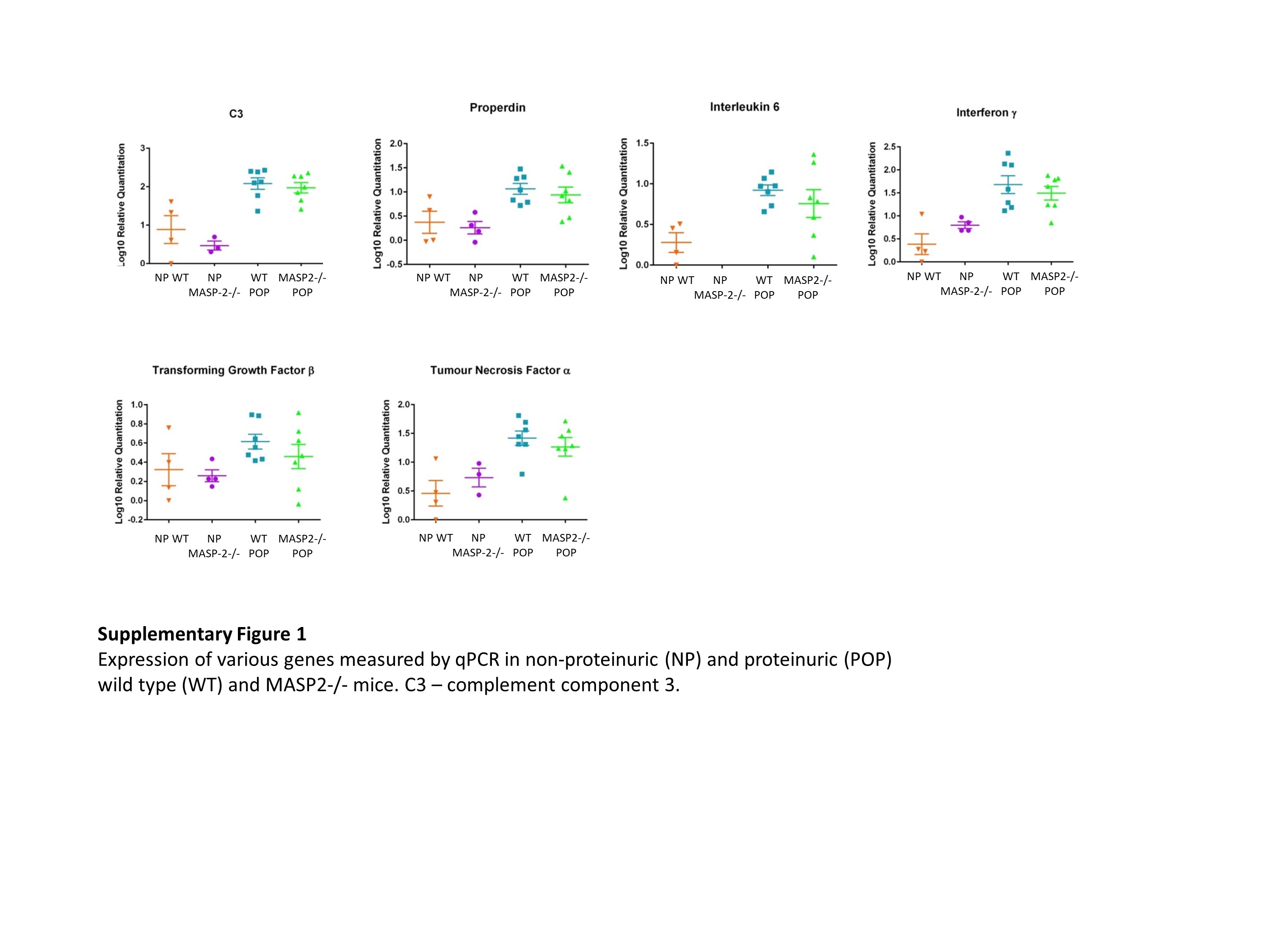

Supplement: Supplementary file 1 [file Image_1.JPEG]

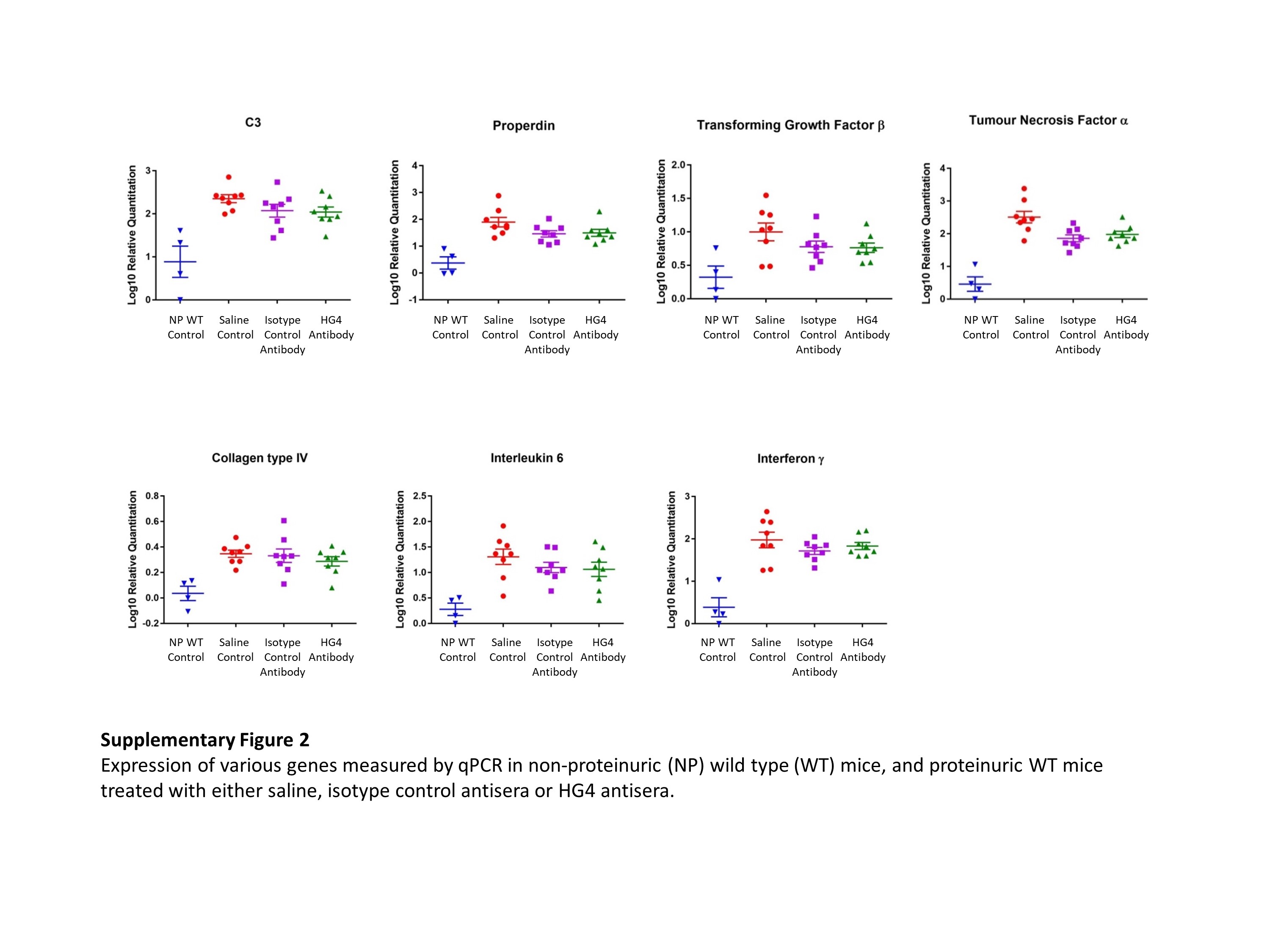

Supplement: Supplementary file 2 [file Image_2.JPEG]
